# Pastoral subsistence and mounted fighting in the Eastern Tianshan Mountain region: New insights from the Shirenzigou worked bone assemblage

**DOI:** 10.1371/journal.pone.0259985

**Published:** 2021-12-14

**Authors:** Yue Li, Chengrui Zhang, Zexian Huang, Huan Liu, Meng Ren, Tongyuan Xi, Jian Ma, Jianxin Wang

**Affiliations:** 1 School of Cultural Heritage, Northwest University, Xi’an, China; 2 Collaborative Research Center on Silk Road Archaeology, Northwest University, Xi’an, China; 3 Ministry of Science and Technology “One Belt, One Road” Joint Laboratory of Human and Environment in China and Central Asia, Northwest University, Xi’an, China; 4 Department of Anthropology, Harvard University, Cambridge, MA, United States of America; 5 School of Resource, Environment, and Historical Culture, Xianyang Normal University, Xianyang, China; New York University, UNITED STATES

## Abstract

Situated at a geographic crossroads, the eastern Tianshan Mountain region in northwest China is crucial to understanding various economic, social, and cultural developments on the Eurasian Steppes. One promising way to gain a better knowledge of ancient subsistence economy, craft production, and social change in the eastern Tianshan Mountain region is to study the artifact assemblages from archaeological contexts. Here, we present an analysis of 488 worked animal bones from the large site of Shirenzigou (ca. 1300–1 BCE), to date the largest assemblage of this kind uncovered in the eastern Tianshan Mountain region. We classified these worked bones into six categories, including “ritual objects”, “ornaments”, “tools”, “worked astragali”, “warfare and mobility”, and “indeterminate”. The identification of animal species and skeletal elements indicates that worked bones from Shirenzigou are characterized by a predominance of caprine products, particularly worked astragali, which is consistent with the large proportion of caprine fragments found in animal remains associated with food consumption. This demonstrates the contribution of caprine pastoralism to bone working activities at Shirenzigou. The making of most worked bones does not appear to have required advanced or specialized skills. Considering the absence of dedicated bone working space, alongside the variability in raw material selection and in dimensions of certain types of artifacts, we infer that worked bone production at Shirenzigou was not standardized. In terms of raw material selection and mode of production, Shirenzigou differed from their settled, farming counterparts in the Yellow River valley of northern China. In addition, along with the evidence for violence and horseback riding, the increasing use of bone artifacts associated with warfare and mobility during the late occupation phase of Shirenzigou reflects growing social instability and implies the likely emergence of single mounted horsemen, equipped with light armors, in the region during the late first millennium BCE. Our results provide new insights into animal resource exploitation and changing lifeways of early pastoral societies in the eastern Tianshan Mountain region, expanding our knowledge of the economic, social, and political milieu of late Bronze Age and early Iron Age eastern Eurasia.

## Introduction

The second and first millennium BCE witnessed the movement of human populations, the dispersal of animal and plant domesticates, the transmission of technologies, and the formation of trade networks across the Eurasian Steppes [1–9]. These transregional exchanges, facilitated in part by the use of domestic horses for transport, brought in new changes to subsistence economy, administrative organization, and military strategy, having profound influences on the following course of Eurasian history [10–19].

The eastern Tianshan Mountain region, encompassing present-day Hami and adjacent areas in Xinjiang, northwest China, is located at a crossroads in eastern Eurasia. Over millennia, it has been a channel for extensive east-west interactions involving populations of diverse backgrounds [20–29]. The westward spread of painted ceramics from the Hexi Corridor, the cultural influence of the Andronovo horizon from the far west, to name but a few, have all been documented in the archaeological record of the eastern Tianshan Mountain region [30–31]. The unique geographic position and environmental conditions make the region particularly crucial to understanding various transformations in ancient eastern Eurasia.

The study of artifacts has proved to be a key perspective, from which we can have a better knowledge of subsistence strategy, exchange, and social organization in ancient societies [32]. Previous analysis of artifacts from archaeological contexts, such as gold, iron, and glass products, has shed some light on early craft production and cultural communications in the eastern Tianshan Mountain region [33–38]. Nevertheless, the artifacts examined in these insightful studies were primarily from mortuary contexts dated to the late first millennium BCE, and the sample size involved was relatively small. To some extent, this prevents a more comprehensive understanding of the broader implications of artifact assemblages for the economic and social milieu of ancient eastern Tianshan Mountain region.

Here, we present an analysis of worked animal remains from the large site of Shirenzigou in the eastern Tianshan Mountain region, namely animal hard tissues (e.g., bones, antlers, and teeth) that were morphologically modified and utilized by humans to meet a variety of demands [39–45], hereafter referred to as “worked bones”. Previous years of fieldwork at Shirenzigou has uncovered a large number of worked bones from a wide range of archaeological features dated to the late second and first millennium BCE. Applying zooarchaeological methods, we examine the animal species and skeletal elements chosen for making these worked bones, the mode of production, and chronological variations in the use of worked bones at Shirenzigou. Alongside other relevant lines of archaeological evidence, we explore the implications of the Shirenzigou worked bone assemblage for subsistence economy, craft production, and social development in the eastern Tianshan Mountain region and beyond during the late Bronze Age and early Iron Age.

### The site of Shirenzigou

**Fig 1:**
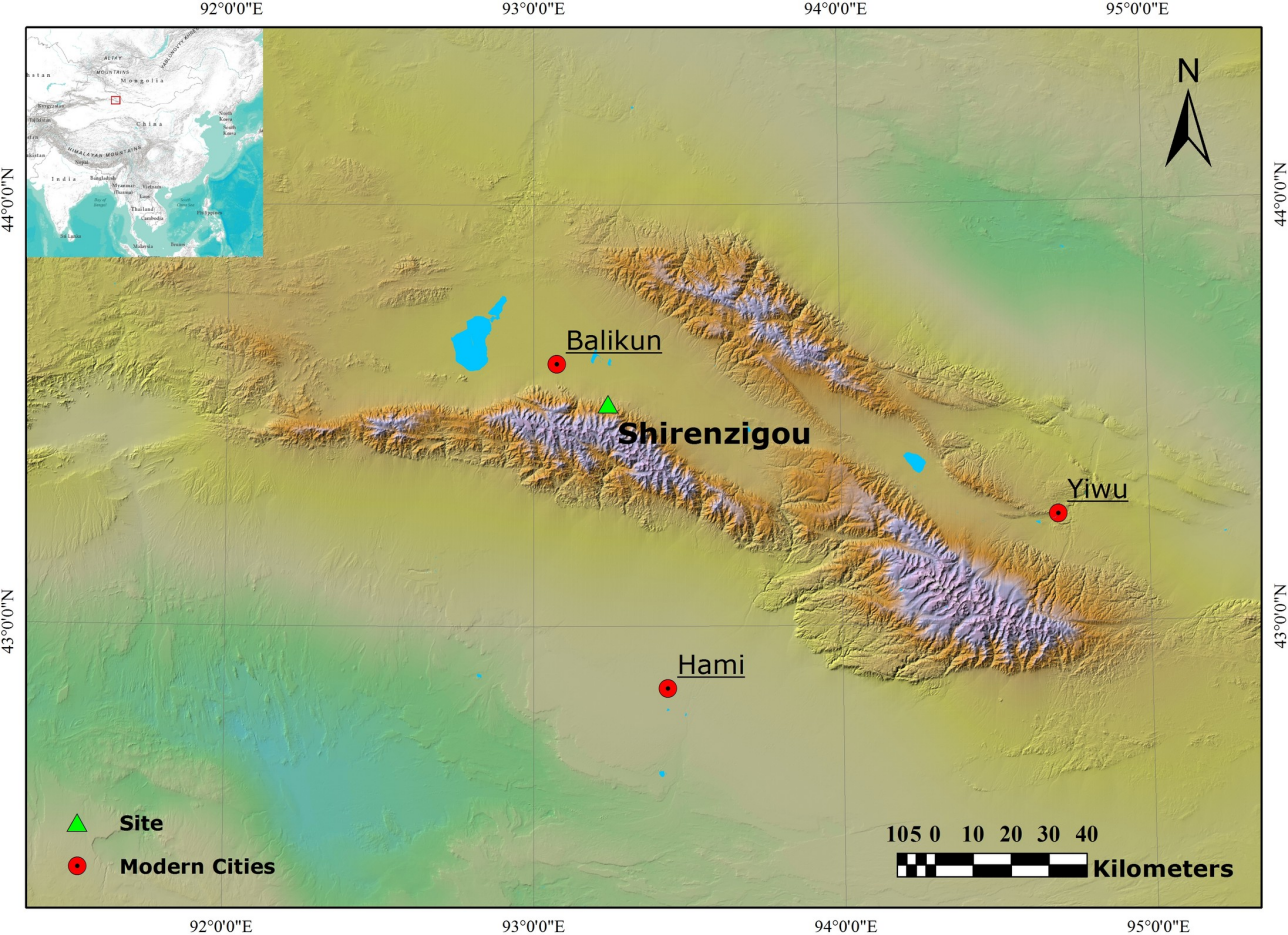
Map showing the location of Shirenzigou in the eastern Tianshan Mountain region, northwest China. Map produced in ArcMap 10.7.1. The digital elevation model was acquired from the Geospatial Data Cloud site, Computer Network Information Center, Chinese Academy of Sciences (http://www.gscloud.cn).

**Fig 2:**
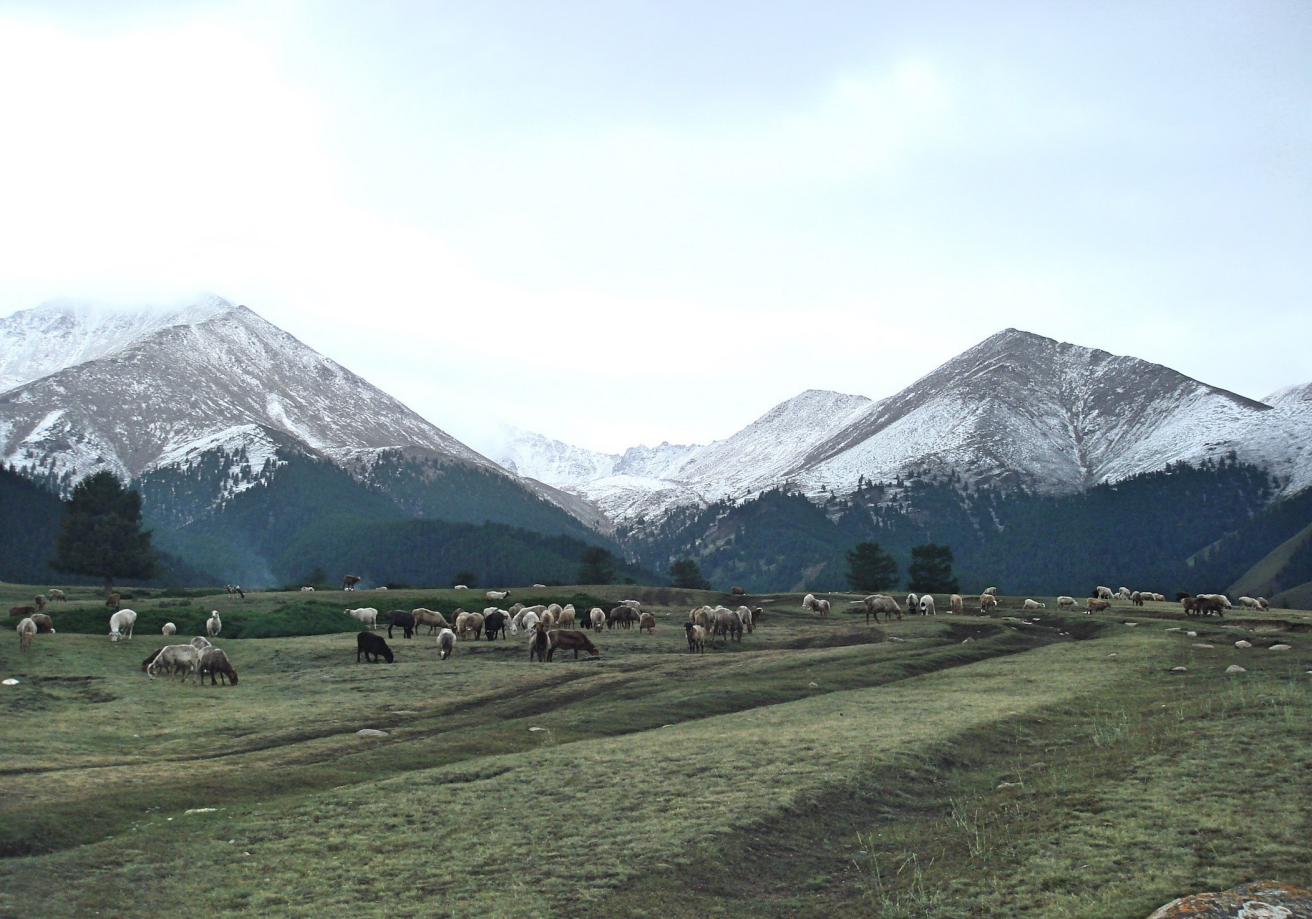
Photo showing modern natural landscape surrounding the site of Shirenzigou.

Shirenzigou (43°31′12.8′′-43°34′28.9′′N, 93°13′44.8′′-93°16′49.1′′E), previously known as Dongheigou, is located north of the eastern section of the Tianshan Mountain range, near modern Shirenzi village in the county of Balikun, Xinjiang **(Figs 1 and 2)**. Natural landscape surrounding Shirenzigou is characterized by moraine hills and alluvial fans covered with mountain meadows and gravels [46]. The site was first discovered in 1957 during the general survey of cultural relics in the Hami region [47]. In the summer of 2005, the School of Cultural Heritage at Northwest University and partner institutions conducted systematic field survey on the site and its environs. Results show that archaeological features are widely distributed in an area of approximately 8.8 hectares, suggesting that Shirenzigou is one of the largest sites in the eastern Tianshan Mountain region [48, 49].

Excavations in the following years revealed a large quantity of features, including, but not limited to mound-platforms, architectural structures, burials, and sacrificial pits, along with abundant artifacts of various materials [47, 48, 50]. The large stone mound-platforms are located in the southern part of the site, from which wooden walls, postholes, hearths, and pits of varying functions were uncovered. The location, size, and structure of these mound-platforms indicate their importance at Shirenzigou. Despite differences in size and mortuary objects, burials at the site are similar in structure, characterized by an above-ground stone mound, a vertical pit, and a burial chamber dug into the pit. Some burials have affiliated pits with animal deposits [47, 51, 52]. The configuration of burials and the decorative design of certain artifacts point to cultural communications between the eastern Tianshan Mountain region and other parts of the Eurasian Steppes, such as the Altai Mountains [27].

**Fig 3:**
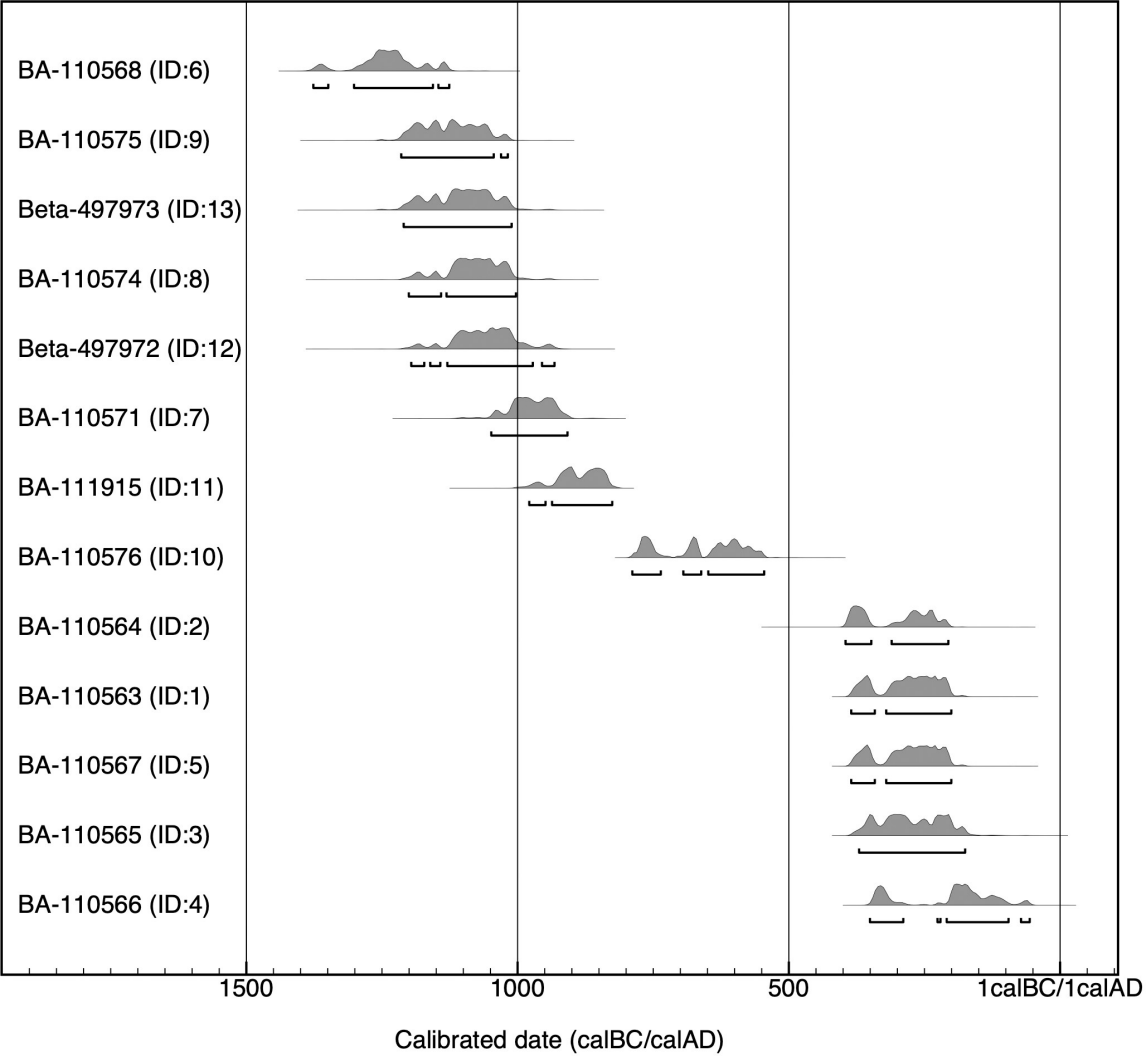
Calibrated radiocarbon dates for Shirenzigou. Conventional radiocarbon ages were generated by the Accelerator Mass Spectrometry Dating Laboratory of Peking University in Beijing. Calibration was performed in OxCal v4.4 [53], using IntCal20 as the calibration curve [54] (https://c14.arch.ox.ac.uk/oxcal/OxCal.html). Most radiocarbon dates in this figure (IDs 1–8, 11–13) were reported in previous research [55–57], and in this study we recalibrated these data using the most recent calibration curve (**S1 Table**).

Based on seriation of artifacts and archaeological features, along with direct radiocarbon dates derived from animal bones and plant remains, the occupation of Shirenzigou can be divided into three phases, spanning over one millennium **(Fig 3)**. The early phase covered a period from ca. 1300 BCE to ca. 800 BCE. The middle and late phases spanned the following 800 years, demarcated in ca. 500–400 BCE.

## Materials and methods

In this study, we analyzed an assemblage of 488 worked bones. These specimens were collected during excavation in previous years (2006–2012) and are currently stored in Hami Museum. A total of 429 worked bones were unearthed from non-mortuary contexts, such as mound-platforms, houses, pits, and cultural layers. The other 59 specimens were from burials. Of 385 worked bones with preliminary chronological estimates, 97 were uncovered from contexts dated to the early occupation phase, 3 the middle occupation phase, and 285 the late occupation phase. This seemingly dramatic fluctuation in the sample size between occupation phases of Shirenzigou, in fact, reflects differences in the number of contexts and their types. Most burials found at Shirenzigou were dated to the late occupation phase. The number of late contexts is also greater than that of earlier ones. Only a tiny portion of contexts were associated with the middle occupation period.

Analysis of the worked bone assemblage was carried out in Hami Museum and the School of Cultural Heritage at Northwest University. We observed these worked bones for taphonomic effects commonly seen in the archaeological record to evaluate their preservation conditions [58–60]. The identification of animal species and skeletal elements was based on published references (e.g., [61, 62]) and comparative animal bone collections, both archaeological and modern, currently housed in the School of Cultural Heritage at Northwest University. We identified each worked bone into the lowest identifiable taxonomic unit. Specimens that could only be identified into size classes (e.g., “large mammal”) due to alteration or loss of distinguishable anatomical traits were grouped as “unidentified mammals”.

Although the morphology of a given bone artifact does not necessarily reflect its function, and one bone artifact may have been used for different purposes [63], we categorized worked bones from Shirenzigou based on inferences of their possible functions from a series of factors, such as shape, dimension, use wear, and ethnographic information. The close-ups of worked bones were photographed using a NIKON SMZ25 Research Stereo Microscope.

## Results

### Preservation of worked bones

We identified limited post-depositional effects on worked bones from Shirenzigou (S2 Table). About 4.7% (n = 23) of worked bones exhibit traces of weathering, but the degree is generally low. Rodent gnawing and carnivore chewing occurred on 6.1% (n = 30) of all specimens. Traces of burning were recorded for a small portion of worked bones (n = 32, 6.6%). These data suggest that the worked bone assemblage examined was basically well preserved.

### Categories of worked bones

**Fig 4:**
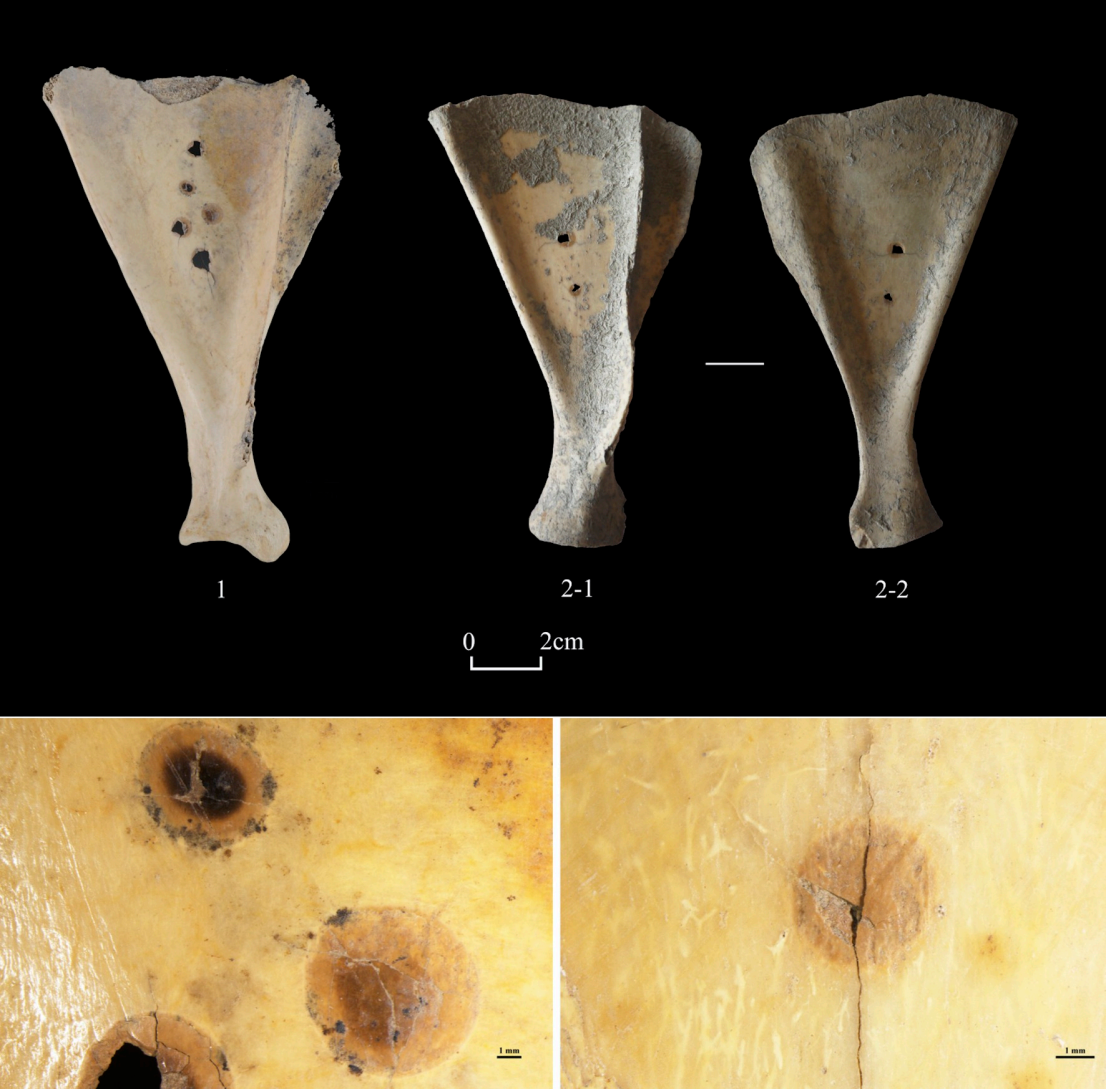
Examples of oracle bones from Shirenzigou. 1) specimen from 2009HBSIIIT0822④; 2) specimen from 12XBSIIIT0720II-3④. The close-ups at the bottom show brownish burned marks on the scapulae (Left: specimen from 2009HBSIIIT0822④; Right: specimen from 2011XBSIIIHD12).

We classified the 488 worked bones into six categories, namely “ritual objects”, “ornaments”, “tools”, “worked astragali”, “warfare and mobility”, and “indeterminate” **(S3 Table)**. The category of “ritual objects” contains 6 worked scapulae, unearthed from non-mortuary contexts, taking up 1.2% of all worked bones examined. We identified traces of grinding on 2 scapulae, of which the spine of one was deliberately removed. There is little evidence for pre-treatment (e.g., flattening) of the other 4 specimens. A total of 5 scapulae exhibit round, brownish burning marks, some of which were burned through **(Fig 4)**. No specimens were drilled or chiseled before burning. These worked scapulae were oracle bones made for ritual purposes, similar to those commonly used for divination in Neolithic and Bronze Age societies in northern China [64].

**Fig 5:**
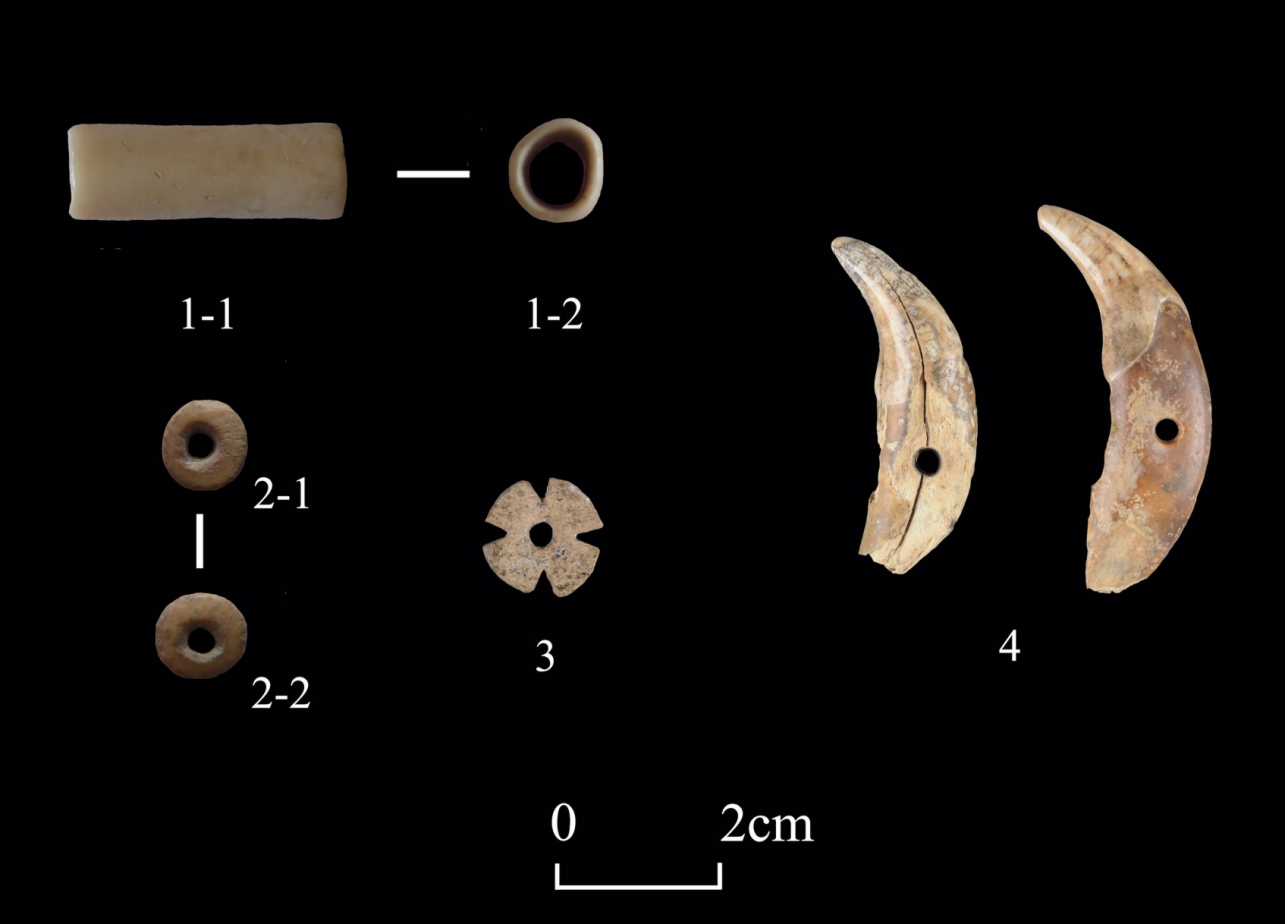
Examples of ornaments from Shirenzigou. 1) specimen from 12XBSIIIT0721I-3④; 2) specimen from 12XBSIIIT0721II-2JX6②; 3) specimen from 2010HBSⅢT0619Ⅲ②; 4) specimen from 2007BSDM016.

About 4.7% (n = 23) of worked bones fall into the category of “ornaments”, which were used primarily for aesthetic or attractive purposes **(Fig 5)**. Some were likely pendants, such as well-ground, perforated carnivorous canines. Other specimens in this category, such as beads, tubes, and small carved chips, were either decorative accessories in themselves or components of larger ornaments.

**Fig 6:**
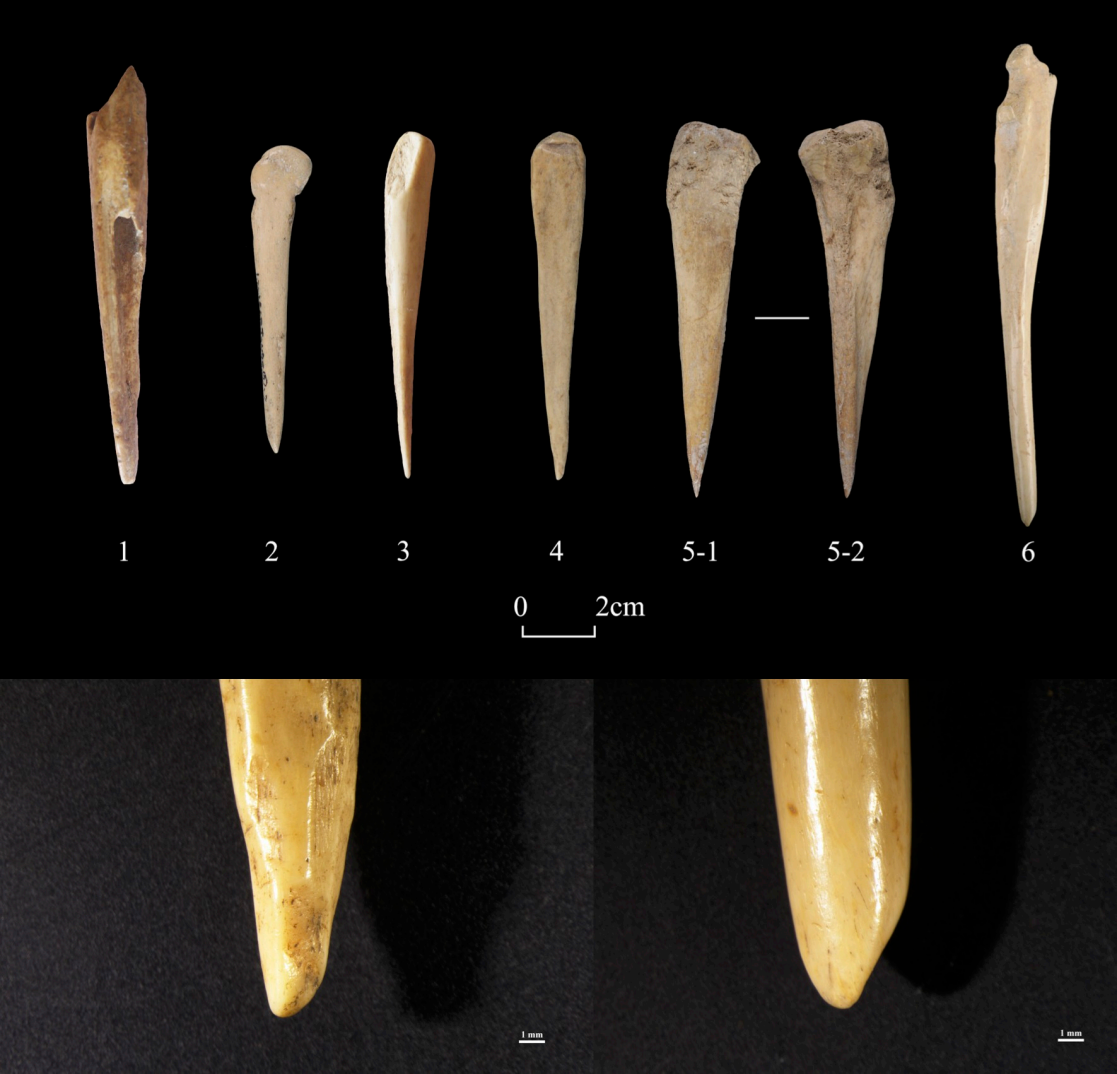
Examples of bone awls from Shirenzigou. 1) specimen from 12XBSIIIH015; 2) specimen from 2010HBSIIIT0619III②; 3) specimen from 12XBSIIIF003; 4) specimen from 2009HBSIIIT0719①; 5) specimen from 11XBSIIIT0720IV-2②; 6) specimen from 2010HBSIIIT0720I③. The close-ups at the bottom show polished tips of the awls (Left: specimen from 2009HBSIIIT0719①; Right: specimen from 2010HBSIIIT0720I③).

**Fig 7:**
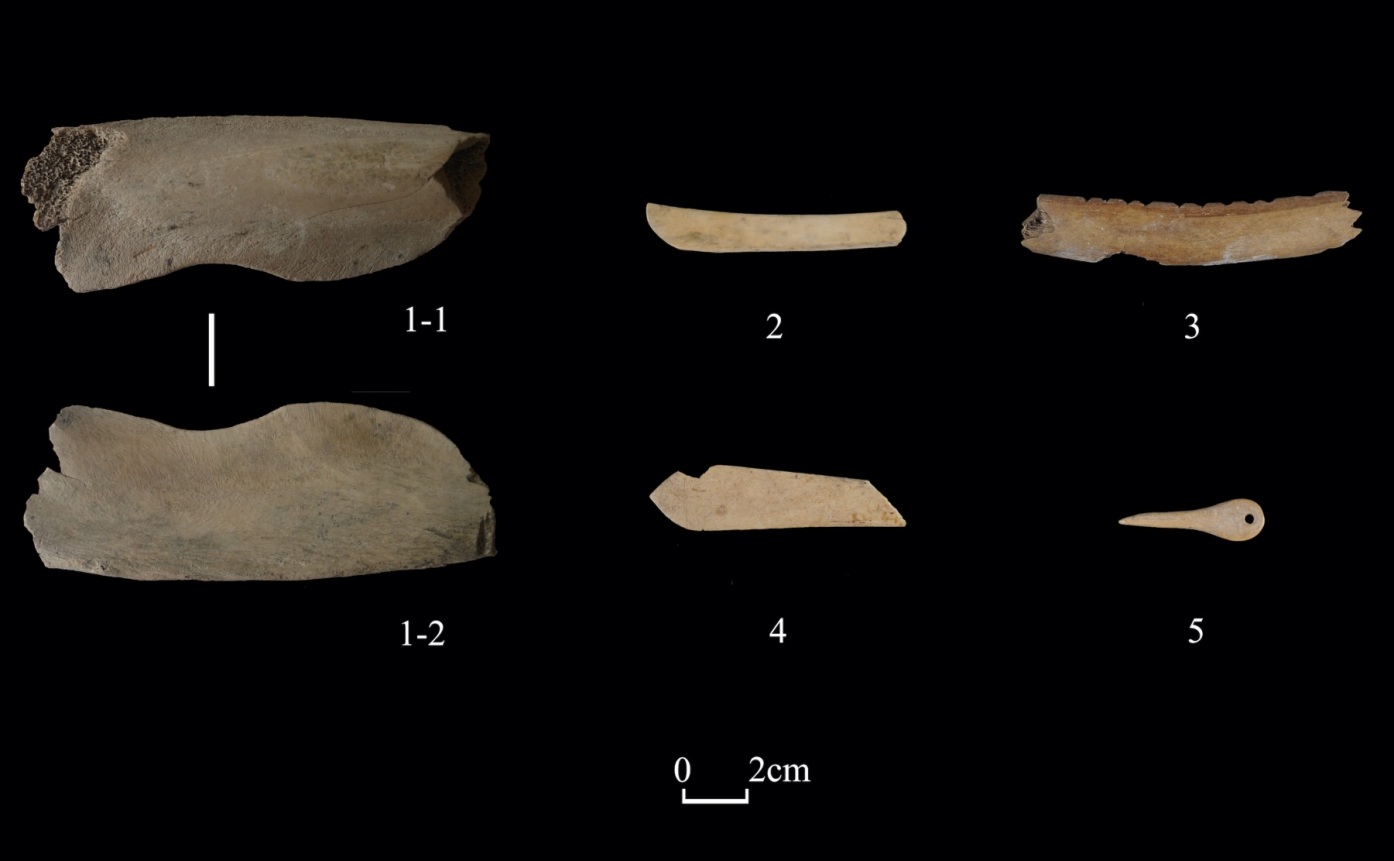
Examples of bone tools from Shirenzigou. 1) specimen from 12XBSIIIT0720II-2③; 2) specimen from 2009HBSIIIT0822④; 3) specimen from 2010HBSIIIT0819IV①; 4) specimen from 2010HBSIIIT0720I②; 5) specimen from 2011XBSIIIDM4.

“Tools” (n = 42) constitute 8.6% of worked bones from Shirenzigou. These artifacts were made for practical purposes (e.g., manufacture). Awls (n = 31) are the largest proportion of tools. Uncovered predominantly from domestic contexts, these undecorated awls were ground and polished **(Fig 6)**. The length of 25 awls with available measurements ranges from 44.03mm to 126.10mm, with an average of 89.52mm (standard deviation: 22.82). Other tools, such as spatula, knife, shovel, and pin, were also commonly seen in previous archaeological record, and the total number of these artifacts is small (n = 11) **(Fig 7)**.

**Fig 8:**
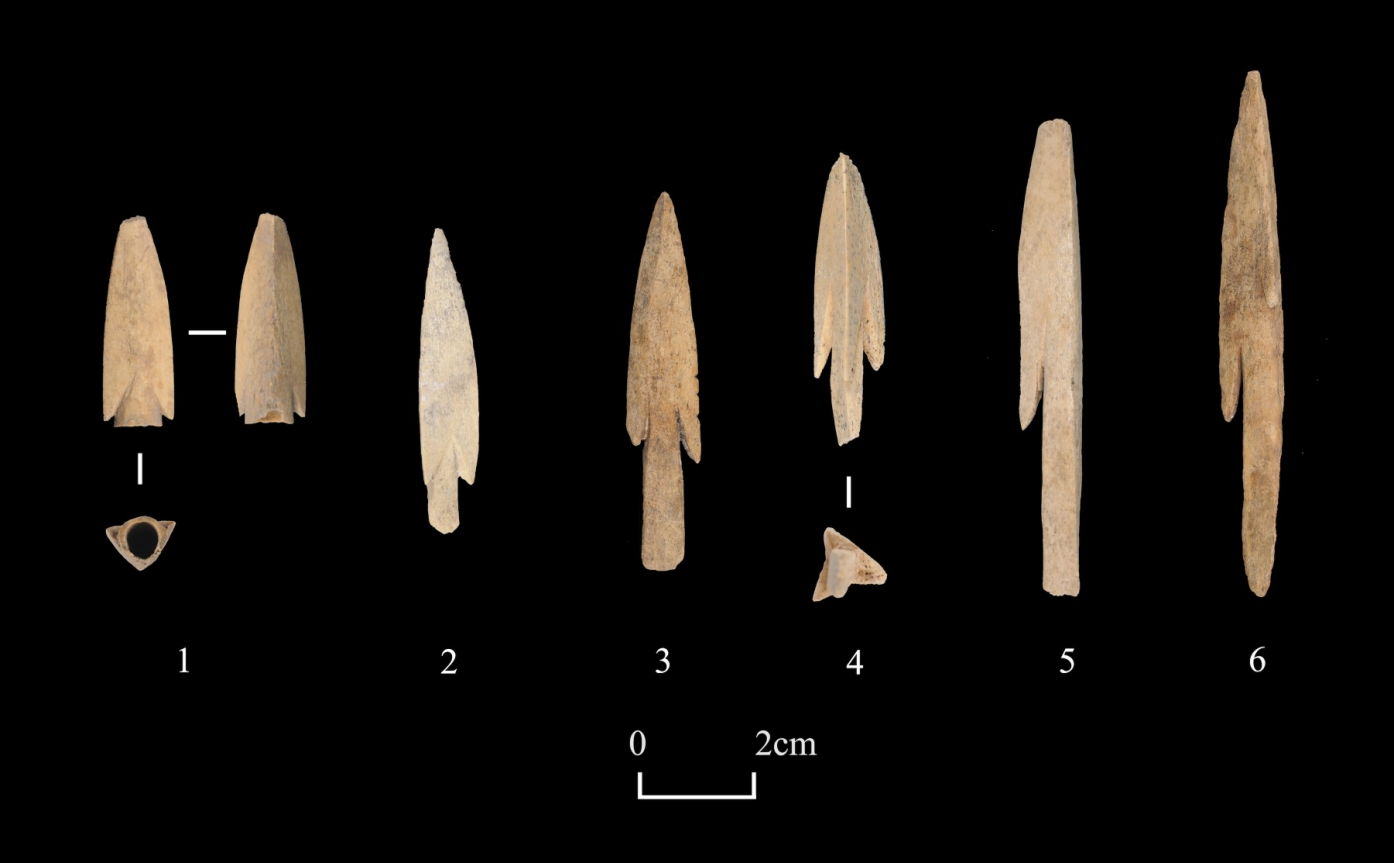
Examples of bone arrowheads from Shirenzigou. 1) specimen from 2009HBSIIIT0719③; 2) specimen from 2010HBSIIIM003④; 3) specimen from 2007BSDIVM013; 4) specimen from 2010HBSIIIM003⑤; 5) specimen from 2010HBSIIIM003④; 6) specimen from 2007BSDIVM013.

Artifacts in the category of “warfare and mobility” (n = 67) comprise 13.7% of all worked bones analyzed. Arrowheads (n = 38) make up the largest proportion of specimens in this category, of which nearly two thirds were from mortuary contexts. A total of 29 arrowheads were either double-winged or single-winged, while the other 9 were wingless, three-winged, or incomplete specimens **(Fig 8)**. The body of some arrowheads was decorated with superficial carved lines. In total, 30 well-preserved specimens have measurements of the full length, the value of which ranges from 30.00mm to 92.48mm, with an average of 62.19mm (standard deviation: 16.74). A small portion of specimens (n = 6) in this category were associated with horse transport, including fittings used to tie reins (similar to the function of buckles) and rod-shaped cheekpieces.

**Fig 9:**
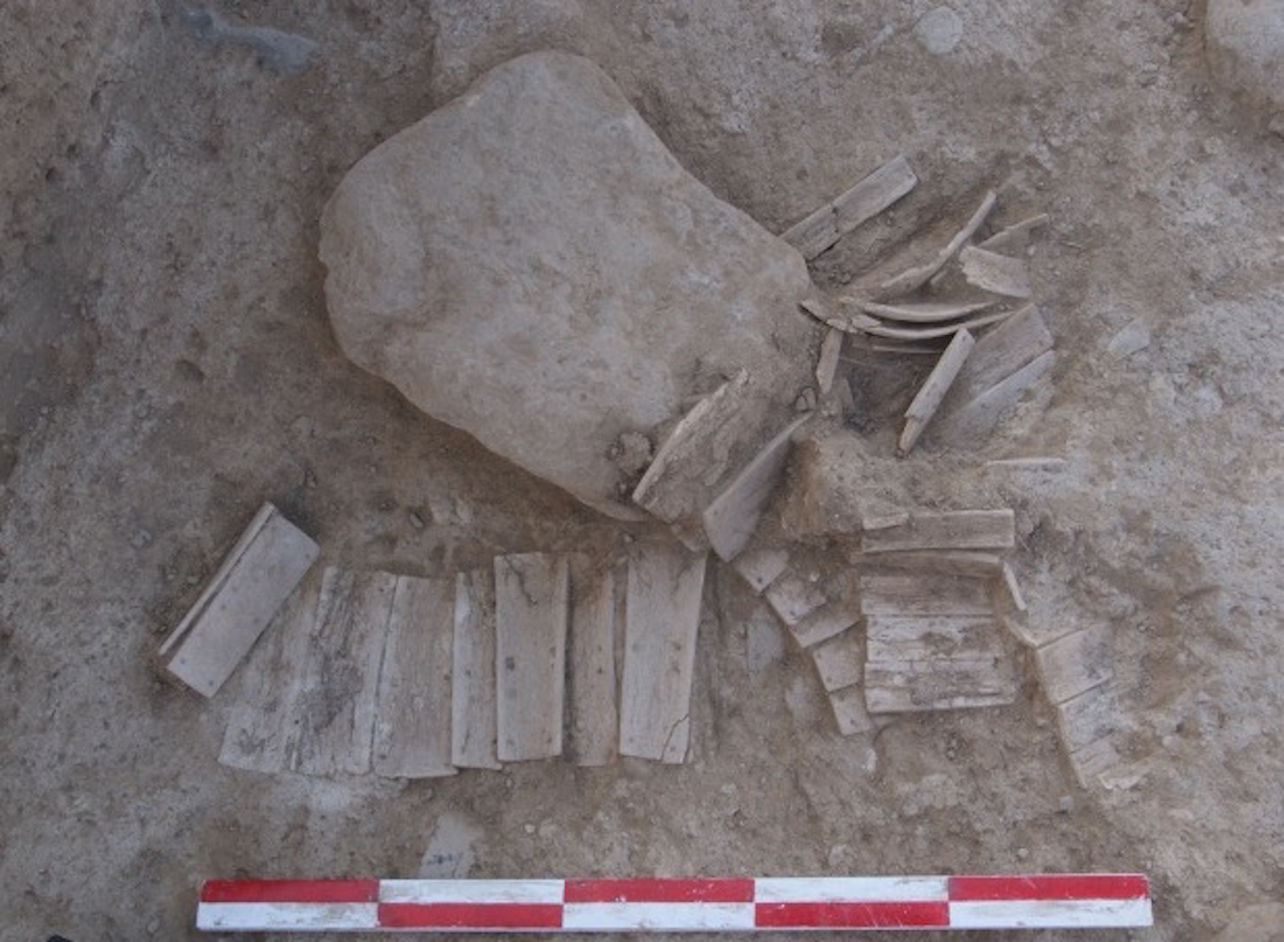
Bone plates unearthed from house F002 at Shirenzigou.

**Fig 10:**
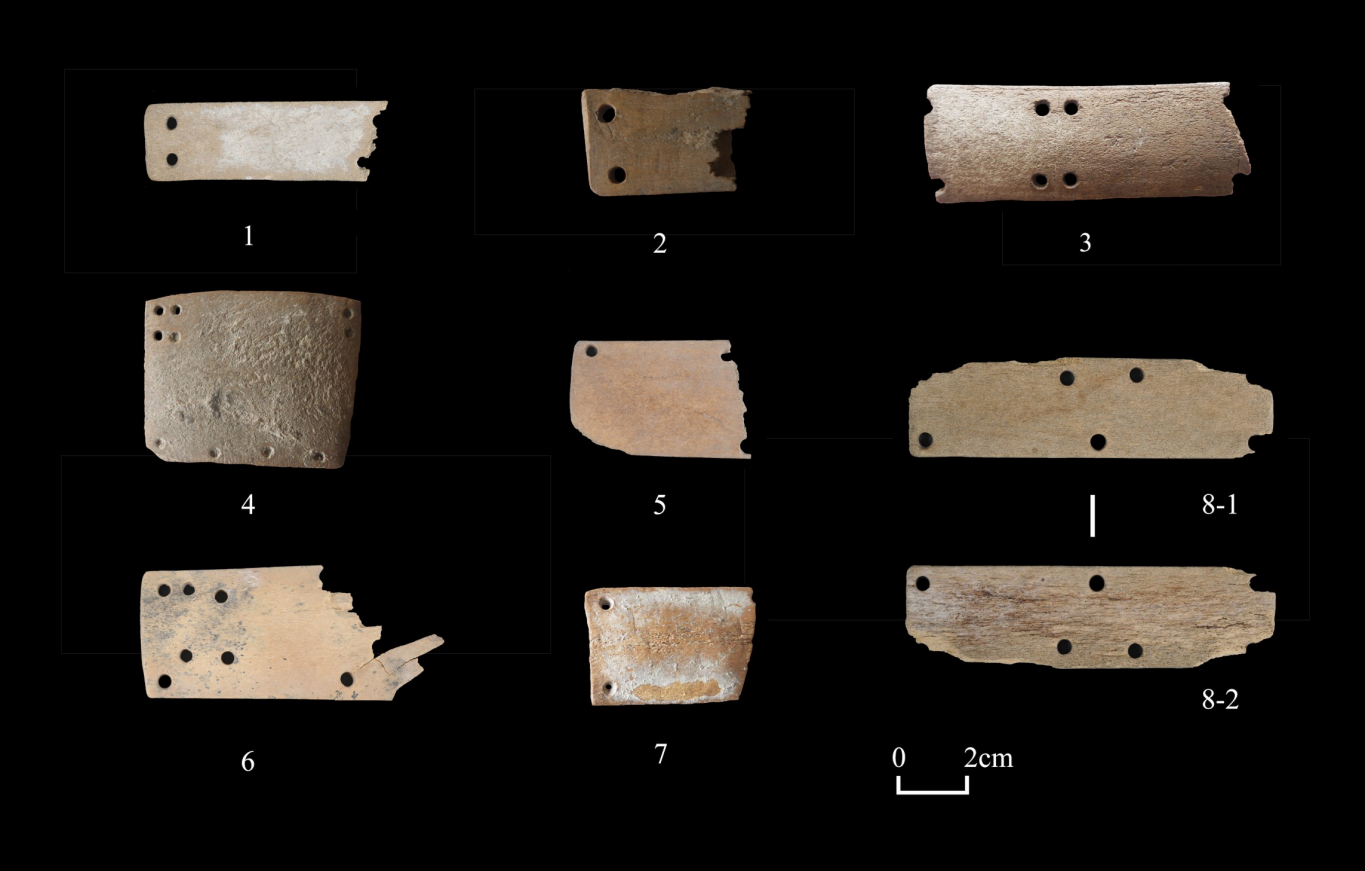
Examples of bone plates from Shirenzigou. 1) specimen from 2010HBSIIIT0620II②; 2) specimen from 12XBSIIIT0720IIIH025; 3) specimen from 12XBSIIIT0721II-2②; 4) specimen from 12XBSIIIT0720IIIH020; 5) specimen from 2010HBSIIIT0620II③; 6) specimen from 2010HBSIIIF004④; 7) specimen from 12XBSIIIF006; 8) specimen from 2010HBSIII-P2③.

One special type of worked bones in “warfare and mobility” is bone plate (n = 23). All from non-mortuary contexts, these specimens are rectangular in shape, well cut and ground, exhibiting round perforations in varying numbers **(Figs 9 and 10)**. The full length of 9 nearly complete plates was available for measurement, the value of which ranges from 52.16mm to 87.47mm, with an average of 61.79mm (standard deviation: 13.91). The width of 21 specimens with available measurements ranges from 14.10mm to 49.33mm, with an average of 28.2mm (standard deviation: 9.86). With regard to the form and layout of perforations, these specimens resembled bone plates from mortuary contexts of the first millennium BCE in other parts of northern China. For example, a group of rectangular bone plates were found in the chamber of an elite burial in Beijing, dated to the early Western Zhou period [65]. The average length of these plates is about 73.0mm, and most specimens exhibit at least 10 perforations. Many specimens were aligned and adjoined to form larger pieces, suggesting that these plates were components of an armor [65]. Similar plates of other materials have also been found on the western side of Eurasia, such as those from the Scythian burials of the fifth to fourth centuries BCE in the Middle Don region and from nomadic burials of the early first millennium CE in the Altai Mountains [66, 67]. Analysis has shown that iron plates of this kind were part of light armors for armed warriors [66–68]. It is noted that the 23 bone plates examined in this study only represent a small portion of all similar artifacts found at Shirenzigou. In fact, 71 bone plates were uncovered from architectural structure F002 at the site during the 2009 excavation season [69], but unfortunately, we were not able to analyze these specimens as they are currently under laboratory conservation. Many of these bone plates were aligned in order, with one side of a piece adjoining the opposite side of the neighboring piece, implying that they may have been tied together through the perforations. The layout of F002 and its location at Shirenzigou suggest that it was more likely a sentry than an ordinary residential structure [69]. In light of multiple lines of evidence discussed above, we believe that bone plates found at Shirenzigou were components of light armors for self-protection.

**Fig 11:**
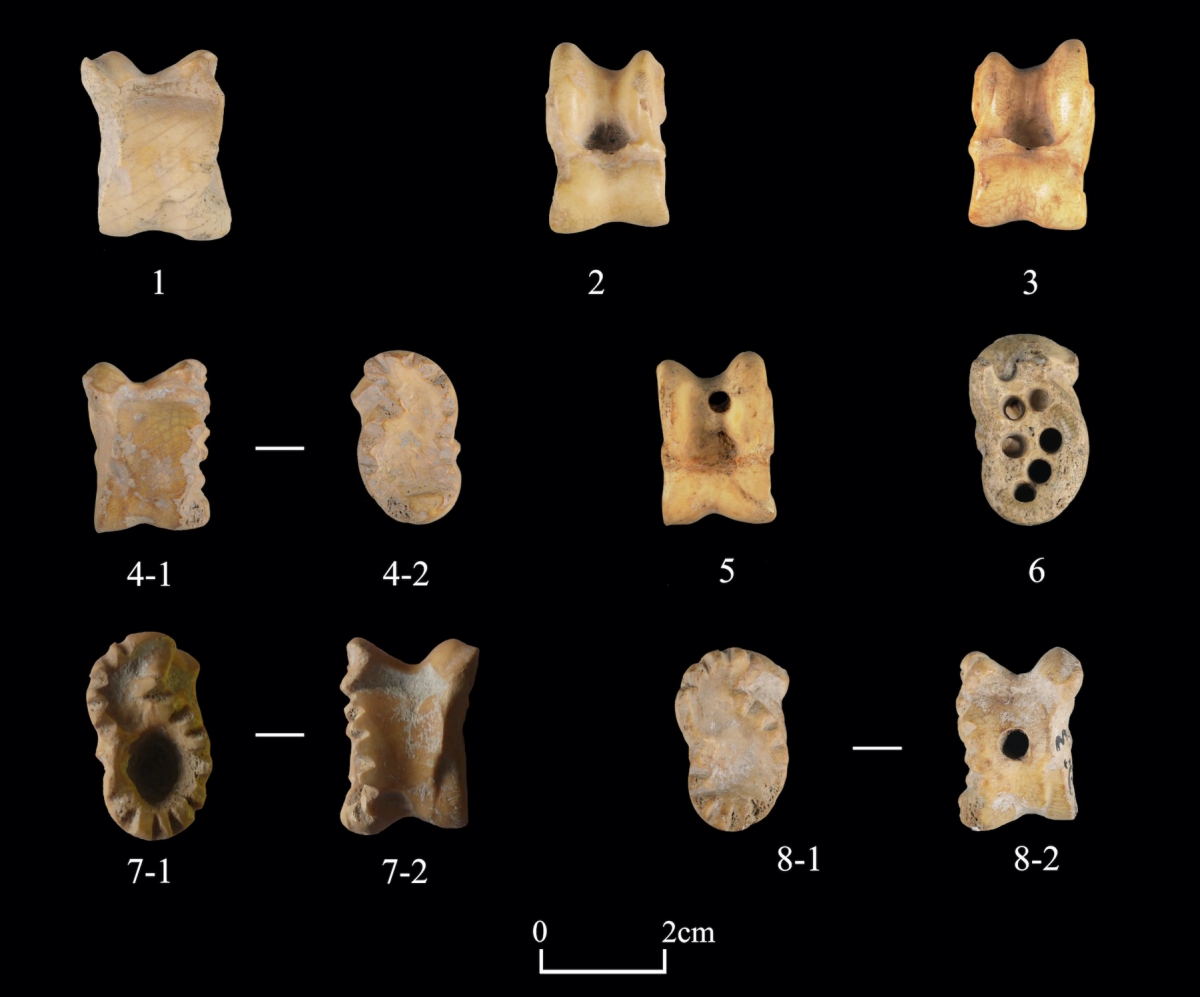
Examples of worked caprine astragali from Shirenzigou. 1) specimen from 2009HBSIIIF002④; 2) specimen from 2009HBSIIIT0719③; 3) specimen from 2010HBSIIIM003②; 4) specimen from 2010HBSIIIT0719IV③; 5) specimen from 2010HBSIIIM003④; 6) specimen from 2010HBSIIIT0720I④; 7) specimen from 12XBSIIIT0720IIIH020; 8) specimen from 2010HBSIIIT0619III②.

**Fig 12:**
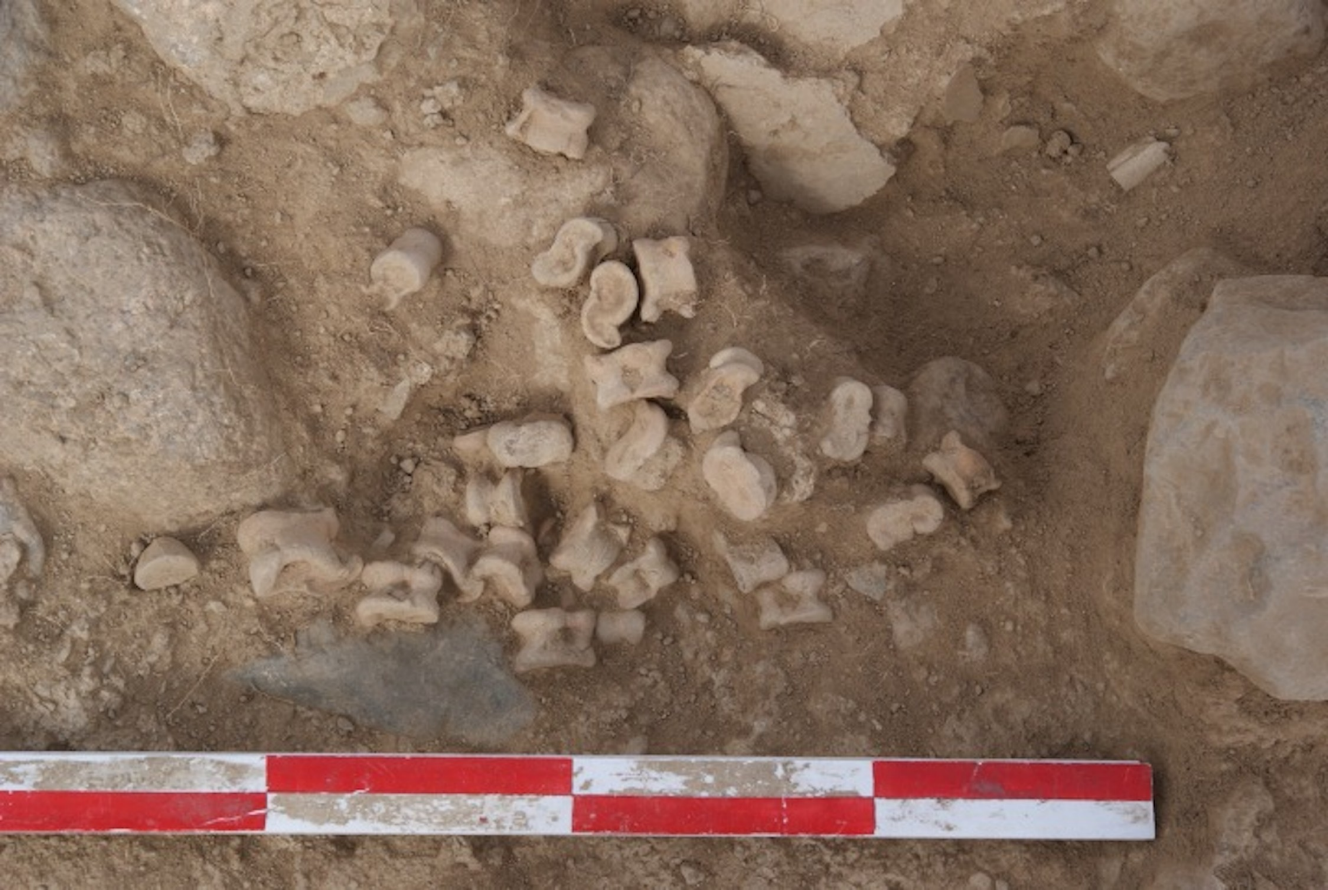
Worked caprine astragali from house F002 at Shirenzigou.

“Worked astragali” (n = 288), taking up 59.0% of the worked bone assemblage, is a unique category. The possible functions of these astragali may have been practical, ritual, or aesthetical, but we could not readily make distinctions. Specimens in this category are primarily caprine astragali (n = 278). While a few were uncovered from mound fillings of two burials, all others were from non-mortuary contexts, commonly found in piles **(Figs 11 and 12)**. The majority of these caprine astragali exhibit smooth, polished surfaces and edges, of which some were carved with superficial lines or decorated with undulating cutmarks. A few specimens have perforations in varying numbers. Previous studies and ethnographic data show that caprine astragali can satisfy a wide range of needs, such as being used as game pieces, dice, or ornaments, and sometimes also for ritual purposes [70, 71]. In the case of Shirenzigou, while most worked caprine astragali may have been used as game pieces, those with perforations were probably decorative objects. It is also likely that some worked caprine astragali (e.g., specimens from burials or completely burned) were associated with ritual practices. Another type of worked bones in this category is slab-like astragali (n = 10). These specimens were predominately made of deer astragalus, the body of which was deliberately cut and ground to form a flat, slab-like cuboid, similar to dominoes in modern societies. Similar to worked caprine astragali, these slab-like astragali were also likely used for recreation or other purposes.

The rest of 62 worked bones are grouped as “indeterminate” (12.7%). Although exhibiting anthropogenic modification traces of varying degrees, such as cutting, sawing, and whittling, these specimens are mostly incomplete and/or in a shape rarely seen in previous archaeological record, making it difficult to characterize their possible functions.

### Raw materials for worked bones from Shirenzigou

**Fig 13:**
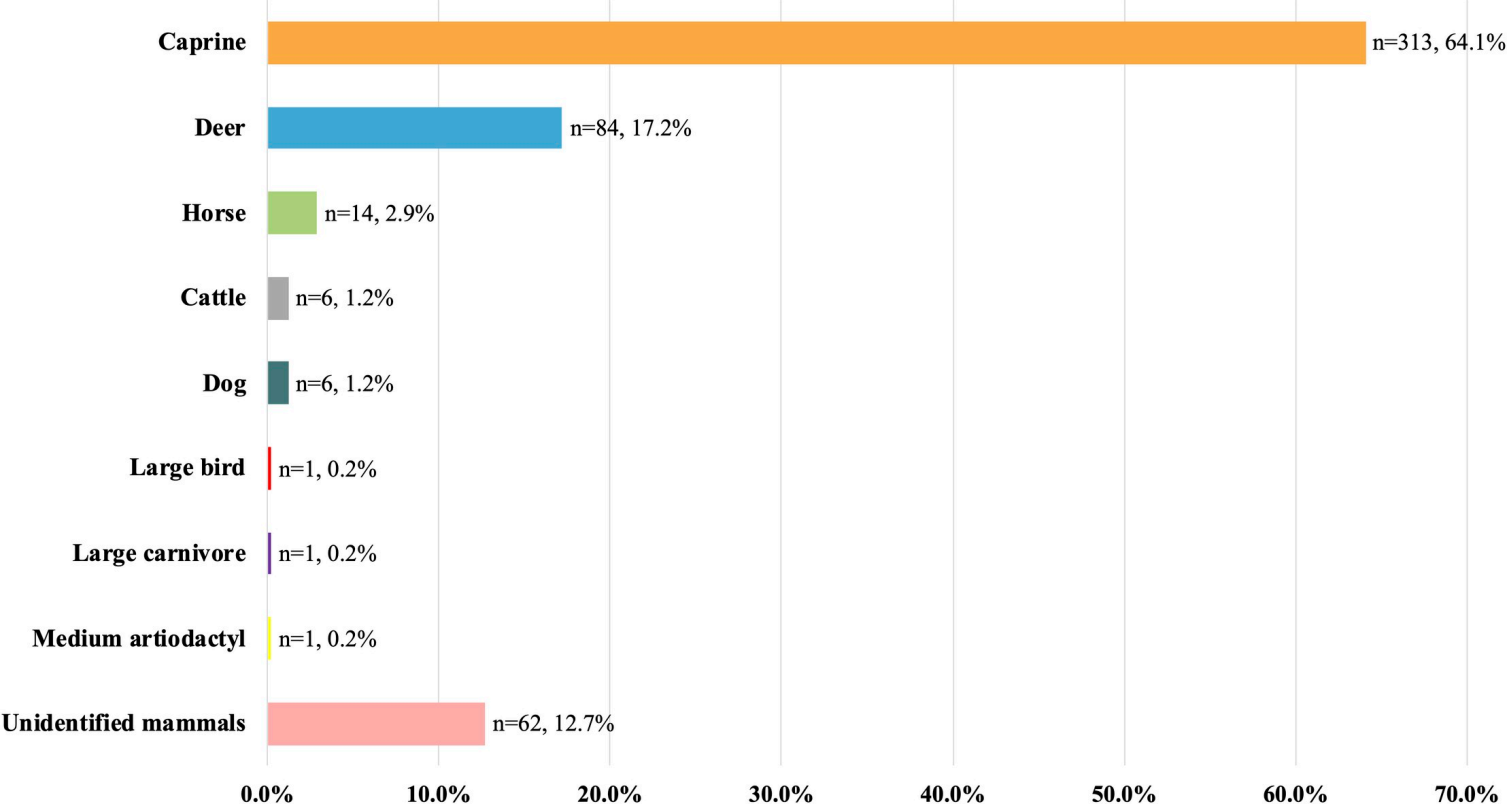
Animal taxonomic structure for worked bones from Shirenzigou.

Our results show that 313 (64.1%) worked bones from Shirenzigou were made of sheep/goats *(Ovis aries/Capra hircus)*, which are grouped into one category as “caprines” **(Fig 13)**. Considering that nearly a quarter of these specimens can be clearly identified as sheep (*Ovis aries*), we infer that the majority of caprine products were made of sheep. This is followed by deer (Cervidae), which take up 17.2% (n = 84) of the whole assemblage. While other animal species, such as horses (*Equus caballus*), dogs *(Canis familiaris)*, and cattle *(Bos taurus)*, were also exploited for producing bone artifacts at Shirenzigou, their contribution was not comparable to that of caprines and deer. About 12.7% (n = 62) of artifacts were made of mammals unidentified into species, of which 50 were mammals of large or medium size.

**Fig 14:**
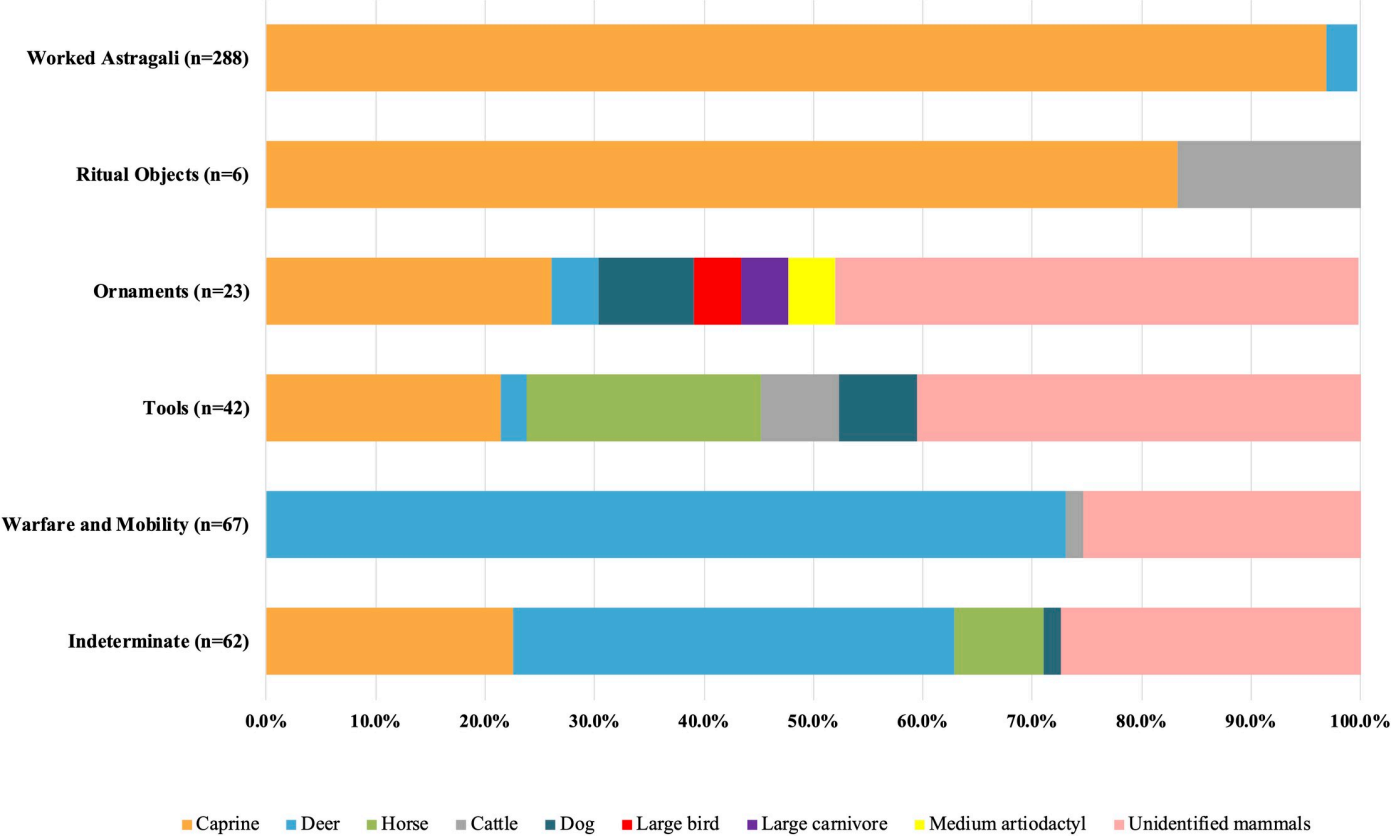
Raw material selection for the six categories of worked bones from Shirenzigou.

In terms of animal species representation for the six categories of worked bones, specimens in “ritual objects” and “worked astragali” are predominately caprine products. As for “tools”, “ornaments”, and “indeterminate”, about a quarter of specimens in each category were made of caprines, only second to unidentified mammals. In contrast, most worked bones in the category of “warfare and mobility” are deer products **(Fig 14)**.

With respect to the selection of skeletal elements, astragali were the most frequently used elements for making bone artifacts, accounting for 59.0% of all worked bones examined. Except for a tiny portion of astragali from deer and cattle, all others were caprine astragali. Antlers (16.8%) rank second, followed by metapodials (3.1%), scapulae (2.7%), ribs (2.5%), tibiae (1.4%), and ulnae (1.2%). All other identified skeletal elements (e.g., femora, mandible, and vertebrae) contribute 3.9% in total. Skeletal elements used for the rest of worked bones can only be classified as “unidentified limb bones” (8.0%) or “unidentified bones” (1.2%). Ritual objects and worked astragali were exclusively made from scapulae and astragali, respectively. Artifacts associated with warfare and mobility were almost exclusively antler (with only a few limb bones) products. A wide range of skeletal elements were used to produce ornaments and tools.

In sum, apart from “indeterminate” worked bones, the choice of raw materials for ornaments and tools appears to have been more variable than that for the categories of “ritual objects”, “worked astragali”, and “warfare and mobility”.

## Discussion

### Early worked bone production in the eastern Tianshan Mountain region

Previous research on bone working practices in Bronze Age and early Iron Age China has primarily focused on the manufacture of bone artifacts in farming societies in the Yellow River valley, a region crucial to understanding early state formation and social transformations in China [72, 73]. By contrast, early bone working activities along the frontier regions, particularly those of pastoral communities, are still poorly characterized. In the case of the eastern Tianshan Mountain region, this is in part due to the rarity of worked bones from archaeological contexts. At the majority of sites in this region excavated prior to Shirenzigou, the number of worked bones unearthed was small (each less than 25), and these artifacts were almost exclusively from mortuary contexts [74–79]. There is also a lack of zooarchaeological analysis of worked bones. Therefore, worked bones from Shirenzigou provide an opportunity to explore animal resource exploitation and craft production in ancient pastoralism-based societies in the eastern Tianshan Mountain region.

At Shirenzigou, caprines (sheep/goats) were the major source of raw materials for worked bone production. Even if we excluded worked caprine astragali (n = 278), caprine products, such as awls, oracle bones, and ornaments, still account for a large proportion of the remaining worked bones (n = 35), only second to deer products (n = 84). Chronologically, worked bones from contexts of both early and late occupation phases of Shirenzigou were characterized by a predominance of caprine products (83.3% and 63.2%, respectively). Even if we did not count in the 288 worked astragali, caprines were also the most frequently exploited domestic animals for worked bone production at Shirenzigou throughout its occupation period. In fact, previous analysis of animal skeletal remains from the residential areas of Shirenzigou, most likely food wastes, has shown that caprine fragments, domestic sheep in particular, constitute approximately 92.3% and 68.0% of animal bones from contexts of the early and late occupation phases of the site, respectively [80]. Together, these data suggest that the pastoral management of caprines played crucial roles in subsistence practices and worked bone production at Shirenzigou. In addition, ethnographic surveys show that modern pastoralists in China, such as those in Inner Mongolia and Xinjiang, still keep the tradition of playing with caprine astragali for leisure [81, 82]. The presence of numerous caprine astragali in worked bone assemblages is also rarely seen in the zooarchaeological record from settled, farming societies in ancient China. These additional lines of evidence highlight the contribution of caprine herding to subsistence strategies by the Shirenzigou occupants.

Except for a tiny number of specimens that were likely production wastes (e.g., discarded blanks or debitage), all worked bones from Shirenzigou appear to be finished products. We did not see complete stages of bone artifact production at the site. There is also no evidence for the presence of specific bone working space at the site. In addition, apart from a few artifacts (e.g., arrowheads), the production of most worked bones did not seem to require high degree of skill proficiency and labor expenditure. For example, over half of awls were made from ulnae and metapodials of horses and dogs, the ends of which were modified into awl tips. Similarly, the making of spatulas expediently took advantage of rib shafts of medium-to-large mammals. The smooth surfaces of worked caprine astragali appear to have been formed by frequent use (i.e., contact with human skin), rather than intentional polishing. Furthermore, raw material selection and dimensions of certain types of worked bones that took up a relatively larger proportion, such as awls and arrowheads, were apparently variable. These observations imply that worked bone production at Shirenzigou was likely not standardized.

Our results from Shirenzigou provide additional insights into worked bone production of pastoral communities and farming populations in northern China during the late second and first millennium BCE. In the case of settled societies in the Yellow River valley, where pig husbandry and millet cultivation were crucial components to subsistence strategies [83], bone artifacts were primarily made of domestic cattle [84–91]. Although zooarchaeological evidence has pointed to an increase in the use of domestic cattle, sheep, and goats for meat and secondary products in this region since the late third millennium BCE [83, 92–94], the role of caprines in worked bone production was not comparable to that of cattle. In terms of capital-level sites contemporary with Shirenzigou, such as Anyang of the Shang Dynasty, Fenghao of the Western Zhou Dynasty, and Xianyang of the Qin State, the manufacture of bone artifacts was carried out in large, dedicated workshops and was characterized by specialization and organization [72, 73, 86–91].

Previous fieldwork has revealed a tiered hierarchy of settlements in relation to scale and intra-site layout in the eastern Tianshan Mountain region during the late Bronze Age and early Iron Age. Large stone architectural structures, elite burials, and elaborate mortuary objects demonstrate that Shirenzigou was a core site of social and political importance among pastoral communities in this region [49, 50, 95]. However, with regard to raw material selection, bone working activities at Shirenzigou stood in stark contrast to those at contemporaneous high-level settlements in the Yellow River valley. This might be explained by differences in factors, such as the nature of subsistence economy, population scale, and demand for bone artifacts, between Shirenzigou and their farming counterparts in the Yellow River valley.

### Early horseback riding and mounted fighting in the eastern Tianshan Mountain region

Historical texts in China, such as *Shiji* (*“Records of the Grand Historian”*) and *Zhanguoce* (*“Intrigues of the Warring States”*), have documented the adoption of mounted horseback riding and associated clothing by settled states in northern China during the Warring States period (ca. the fifth to third centuries BCE) [14, 96–99]. The development and maintenance of cavalry force triggered changes in battlefield tactics, playing crucial roles in remodeling political relations between the rival states and their pastoral neighbors [98, 99]. Despite these textural records, however, archaeological evidence pertaining to the early history of mounted fighting in China remains fragmentary. Our dataset from Shirenzigou, alongside other lines of evidence, provides clues to the emergence of horseback warriors in China’s frontier regions.

Worked bones in the categories of “tools”, “ornaments”, “worked astragali” and “indeterminate” were made and used throughout the whole occupation period of Shirenzigou. However, ritual objects and artifacts in the category of “warfare and mobility” were exclusively uncovered from contexts dated to the late occupation phase, constituting 23.5% of worked bones from contemporary contexts. An increase in the number of worked bones made of deer and horses was also documented for the late occupation phase of the site. The identification of skeletal abnormalities on horses and humans from burials at Shirenzigou and a nearby site Xigou has demonstrated the practice of horseback riding in the eastern Tianshan Mountain region by at least the fourth century BCE [55]. More importantly, the co-occurrence of ridden horses, horse riders, offensive weapons, and defensive armors at Shirenzigou may imply the presence of mounted warriors during the late first millennium BCE. Previous analysis of human skeletal remains from Heigouliang, a burial site contemporary with the late occupation phase of Shirenzigou, has recorded traumatic lesions or wounds resulted from interpersonal violence [100]. In particular, those occurred on lower limb bones (e.g., the distal end of fibula) and the upper portion of the skull (e.g., superciliary arch) likely reflect the elevation difference between combatants, of which one might fight on a mount [100]. This further supports our inference of the practice of mounted fighting in the eastern Tianshan Mountain region at the time.

At Shirenzigou, Xigou, and Tuobeiliang, single horse was found interred in the chambers of burials or affiliated sacrificial pits [55, 75, 101]. Based on this line of evidence, coupled with the variability of bone artifacts associated with horseback riding and violence recorded for Shirenzigou, we infer that the conflicts between peer pastoral communities in the eastern Tianshan Mountain region during the late first millennium BCE may have involved single mounted warriors, who were equipped with light armors. No evidence seems to support the use of these horsemen with fixed order or as an organized military unit.

The latter half of the first millennium BCE saw growing social instability in the eastern Tianshan Mountain region. In some burials at Shirenzigou dated to the late occupation phase, disarticulated human skeletons were found interred with ceramic vessels distinct from those of the burial owners in terms of size, shape, and design [47, 48]. Similar phenomena were also seen in contemporary burials at Heigouliang [102]. These practices of human sacrifice appear to be best explained by increased conflicts between different groups of people. Indeed, a number of arrowheads, horse equipment, and swords were found in those burials of the late first millennium BCE at Heigouliang, echoing the findings from Shirenzigou [102], which stands in contrast with the relatively rarity of violence- and mobility-related artifacts from sites of earlier periods in the same region (e.g., the burial sites of Yanbulake and Baiqier) [74, 78]. The construction of sentry-like stone structures on elevated parts of the site, such as those found at Shirenzigou and Xiagou, also reflects the need for defense [103]. Mounted fighting may have played a crucial role in shaping this social and political landscape of the eastern Tianshan Mountain during the late first millennium BCE. That being said, it remains future fieldwork and comprehensive studies to understand the exact scale and form of early mounted fighting in this region.

## Conclusion

The examination of the large worked bone assemblage from Shirenzigou yields new datasets for evaluating early bone working practices in the eastern Tianshan Mountain region and their economic and social implications. Using zooarchaeological methods, our results demonstrate the crucial roles of caprine pastoralism in worked bone production at Shirenzigou and suggest that the making of worked bones was not standardized. This provides important insights into the differences in raw material selection and mode of production between pastoral societies and contemporary settled, farming populations in the Yellow River valley of northern China during the late second and first millennium BCE. In addition, the co-occurrence of weapons, bone armors, ridden horses, and horse riders at Shirenzigou implies the emergence of mounted fighting in the eastern Tianshan Mountain during the late first millennium BCE, a period crucial to understanding the entanglement of farming populations and pastoral communities along China’s frontiers and the roles of mounted cavalrymen in the geopolitics of China before the establishment of the first united empires by the end of the third century BCE. Overall, our findings from Shirenzigou contribute to a more nuanced narrative of the lifeways of pastoral populations in the eastern Tianshan Mountain region during the Bronze Age and early Iron Age, allowing for a better understanding of the economic, social, and political milieu of ancient eastern Eurasia.

## Supporting information

S1 Table Direct radiocarbon dates for the site of Shirenzigou. (DOCX)

S2 Table Taphonomic effects on worked bones from Shirenzigou. (DOCX)

S3 Table Categories of worked bones from Shirenzigou. (DOCX)
