# Supplementary material for: Pastoral subsistence and mounted fighting in the Eastern Tianshan Mountain region: New insights from the Shirenzigou worked bone assemblage
Source: PLoS One. 2021 Dec 14;16(12):e0259985. doi: 10.1371/journal.pone.0259985 (PMC8670691; doi:10.1371/journal.pone.0259985)
Supplement: S1 Table — (DOCX) [file pone.0259985.s001.docx]

**S1 Table. Direct radiocarbon dates for the site of Shirenzigou.**

| **ID** | **Context** | **Material Dated** | **Lab Code** | **Conventional Age (BP)** | **Cal. BC (95.4%)** | **Reference** |
| --- | --- | --- | --- | --- | --- | --- |
| **1** | M012 | Animal bone | BA-110563 | 2230 ± 25 | 386-342 (21.5%)  321-201 (74.0%) | Li et al. 2020 |
| **2** | M012K3 | Animal bone | BA-110564 | 2265 ± 30 | 396-349 (39.5%)  31-207 (55.9%) | Li et al. 2020 |
| **3** | M001 | Animal bone | BA-110565 | 2205 ± 30 | 371-176 (95.4%) | Li et al. 2020 |
| **4** | M012K2 | Animal bone | BA-110566 | 2150 ± 25 | 351-290 (27.6%)  227-221 (0.6%)  210-96 (64.3%)  73-57 (2.9%) | Li et al. 2020 |
| **5** | M011 | Animal bone | BA-110567 | 2230 ± 25 | 386-342 (21.5%)  321-201 (74.0%) | Li et al. 2020 |
| **6** | GT1H31② | Animal bone | BA-110568 | 3000 ± 25 | 1377-1350 (7.0%)  1302-1157 (82.1%)  1147-1127 (6.3%) | Tian et al. 2021 |
| **7** | GT1H11 | Animal bone | BA-110571 | 2825 ± 25 | 1049-909 (95.4%) | Tian et al. 2021 |
| **8** | GT1H25 | Animal bone | BA-110574 | 2895 ± 25 | 1201-1142 (11.7%)  1132-1004 (83.8%) | Tian et al. 2021 |
| **9** | GT1H19 | Animal bone | BA-110575 | 2925 ± 25 | 1215-1045 (92.8%)  1031-1019 (2.7%) | This study |
| **10** | M016K2 | Animal bone | BA-110576 | 2525 ± 25 | 789-737 (27.6%)  695-662 (17.6%)  649-546 (50.3%) | This study |
| **11** | GT1H19 | Plant seed | BA-111915 | 2755 ± 25 | 979-949 (7.5%  937-826 (88.0%) | Ma et al. 2021 |
| **12** | GT1H19 | Plant seed | Beta-497972 | 2880 ± 30 | 1197-1173 (3.5%)  1162-1143 (2.8%)  1130-973 (85.8%)  956-933 (3.3%) | Tian et al. 2021 |
| **13** | GT1H19 | Plant seed | Beta-497973 | 2910 ± 30 | 1211-1012 (95.4) | Tian et al. 2021 |

**References**

Li Y, et al. Early evidence for mounted horseback riding in northwest China. Proceedings of the National Academy of Sciences of the U.S.A. 2020; 117(47):29569-29576.

Ma Z, et al. Xinjiang Dongtianshan Diqu Balikun Shirenzigou Yizhi Chuliangkeng Fenxi. Disiji Yanjiu. 2021; 41(1):214-223. (in Chinese)

Tian D, Ma J, Ren M, Xi T, Wang J, Zhao Z. Xinjiang Diqu De Zaoqi Damai Shengchan: Laizi Tianshan Beilu Shirenzigou Yizhi De Zhiwu Yicun Zhengju. Zhongguo Nongshi. 2021; 3:44-55. (in Chinese)
