# Supplementary material for: Pastoral subsistence and mounted fighting in the Eastern Tianshan Mountain region: New insights from the Shirenzigou worked bone assemblage
Source: PLoS One. 2021 Dec 14;16(12):e0259985. doi: 10.1371/journal.pone.0259985 (PMC8670691; doi:10.1371/journal.pone.0259985)
Supplement: S2 Table — (DOCX) [file pone.0259985.s002.docx]

**S2 Table. Taphonomic effects on worked bones from Shirenzigou.**

| **Type of Taphonomic Effects** | **Number of Specimens from Domestic Contexts** | **Number of Specimens from Burials** | **Total Number and**  **Percentage** | |
| --- | --- | --- | --- | --- |
| Weathering | 12 | 11 | 23 | 4.7% |
| Carnivore chewing or rodent gnawing | 26 | 4 | 30 | 6.1% |
| Burning | 31 | 1 | 32 | 6.6% |
