# Supplementary material for: Pastoral subsistence and mounted fighting in the Eastern Tianshan Mountain region: New insights from the Shirenzigou worked bone assemblage
Source: PLoS One. 2021 Dec 14;16(12):e0259985. doi: 10.1371/journal.pone.0259985 (PMC8670691; doi:10.1371/journal.pone.0259985)
Supplement: S3 Table — (DOCX) [file pone.0259985.s003.docx]

**S3 Table. Categories of worked bones from Shirenzigou.**

| **Category** | **Type of Worked Bones** | **Number of Specimens** | **Percentage** |
| --- | --- | --- | --- |
| Ritual Objects | Oracle bone | 6 | 1.2% |
| Ornaments | Bead | 3 | 0.6% |
|  | Tube | 7 | 1.4% |
|  | Chip | 6 | 1.2% |
|  | Hairpin | 1 | 0.2% |
|  | Pendant | 6 | 1.2% |
| Tools | Awl | 31 | 6.4% |
|  | Knife | 1 | 0.2% |
|  | Pin | 1 | 0.2% |
|  | Needle | 1 | 0.2% |
|  | Shovel | 1 | 0.2% |
|  | Spatula | 4 | 0.8% |
|  | *Huachi* (likely for wool processing) | 3 | 0.6% |
| Warfare and Mobility | Arrowhead | 38 | 7.8% |
|  | Bone plate | 23 | 4.7% |
|  | Horse equipment | 6 | 1.2% |
| Worked Astragali | Caprine astragali | 278 | 57.0% |
|  | Slab-like astragali | 10 | 2.0% |
| Indeterminate | Indeterminate | 62 | 12.7% |
|  | **Total** | **488** | **99.8%*** |

* Due to rounding, the total percentage is smaller than 100.0%.
